# Diagnostic accuracy of oblique chest radiograph for occult pneumothorax: comparison with ultrasonography

**DOI:** 10.1186/s13017-016-0061-x

**Published:** 2016-01-13

**Authors:** Shokei Matsumoto, Kazuhiko Sekine, Tomohiro Funabiki, Tomohiko Orita, Masayuki Shimizu, Kei Hayashida, Taku Kazamaki, Tatsuya Suzuki, Masanobu Kishikawa, Motoyasu Yamazaki, Mitsuhide Kitano

**Affiliations:** Department of Trauma and Emergency Surgery, Saiseikai Yokohamashi Tobu Hospital, 3-6-1 Shimosueyoshi, Tsurumi-ku, Yokohama-shi, Kanagawa 230-0012 Japan; Department of Emergency Medicine, Saiseikai Central Hospital, 1-4-17 Mita, Minato, Tokyo, 108-0073 Japan; Department of Radiological Technology, Saiseikai Yokohamashi Tobu Hospital, 3-6-2 Shimosueyoshi, Tsurumi-ku,, Yokohama-shi, 230-0011 Japan; Division of Emergency Medicine, Fukuoka City Hospital, 13-1 Yoshizukahonmachi, Hakata-ku, Fukuoka, 812-0046 Japan

**Keywords:** Oblique chest radiograph, Lung ultrasound, Occult pneumothorax, Diagnosis

## Abstract

**Backgraound:**

An occult pneumothorax is a pneumothorax that is not seen on a supine chest X-ray but is detected by computed tomography scanning. However, critical patients are difficult to transport to the computed tomography suite. We previously reported a method to detect occult pneumothorax using oblique chest radiography (OXR). Several authors have also reported that ultrasonography is an effective technique for detecting occult pneumothorax. The aim of this study was to evaluate the usefulness of OXR in the diagnosis of the occult pneumothorax and to compare OXR with ultrasonography.

**Methods:**

All consecutive blunt chest trauma patients with clinically suspected pneumothorax on arrival at the emergency department were prospectively included at our tertiary-care center. The patients underwent OXR and ultrasonography, and underwent computed tomography scans as the gold standard. Occult pneumothorax size on computed tomography was classified as minuscule, anterior, or anterolateral.

**Results:**

One hundred and fifty-nine patients were enrolled. Of the 70 occult pneumothoraces found in the 318 thoraces, 19 were minuscule, 32 were anterior, and 19 were anterolateral. The sensitivity and specificity of OXR for detecting occult pneumothorax was 61.4 % and 99.2 %, respectively. The sensitivity and specificity of lung ultrasonography was 62.9 % and 98.8 %, respectively. Among 27 occult pneumothoraces that could not be detected by OXR, 16 were minuscule and 21 could be conservatively managed without thoracostomy.

**Conclusion:**

OXR appears to be as good method as lung ultrasonography in the detection of large occult pneumothorax. In trauma patients who are difficult to transfer to computed tomography scan, OXR may be effective at detecting occult pneumothorax with a risk of progression.

## Background

A traumatic pneumothorax is a common chest injury in which preventable trauma death can occur if appropriate treatment is delayed. The standard anteroposterior supine radiograph (APXR) is the recommended modality to evaluate trauma patients according to the Advanced Trauma Life Support Course guidelines [1]. However, the APXR fails to diagnose a significant proportion of pneumothoraces in this situation. A pneumothorax identified on a computed tomography (CT) scan that was not seen on a preceding supine APXR is termed an “occult pneumothorax” (OPX) [2]. As CT scans are now frequently used to evaluate trauma patients as part of initial management, OPXs can be detected more easily. OPXs account for an astonishingly high 52 to 63 % of all traumatic pneumothoraces [3–5]. OPXs may become life threatening if tension develops, especially in patients receiving mechanical ventilation [6, 7]. Therefore, CT scans should be performed as soon as practicable. It is of note that CT scans are not always performed on patients in severe states of shocks and those in undeveloped countries.

**Fig. 1:**
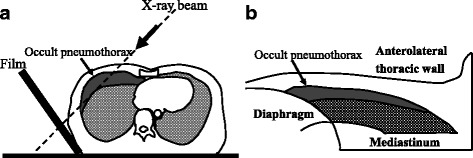
**a** We previously reported a method to detect occult pneumothoraces by supine oblique chest radiography (OXR) without a CT scan. **b** X-ray beam is projected onto a film by this OXR method

**Fig. 2:**
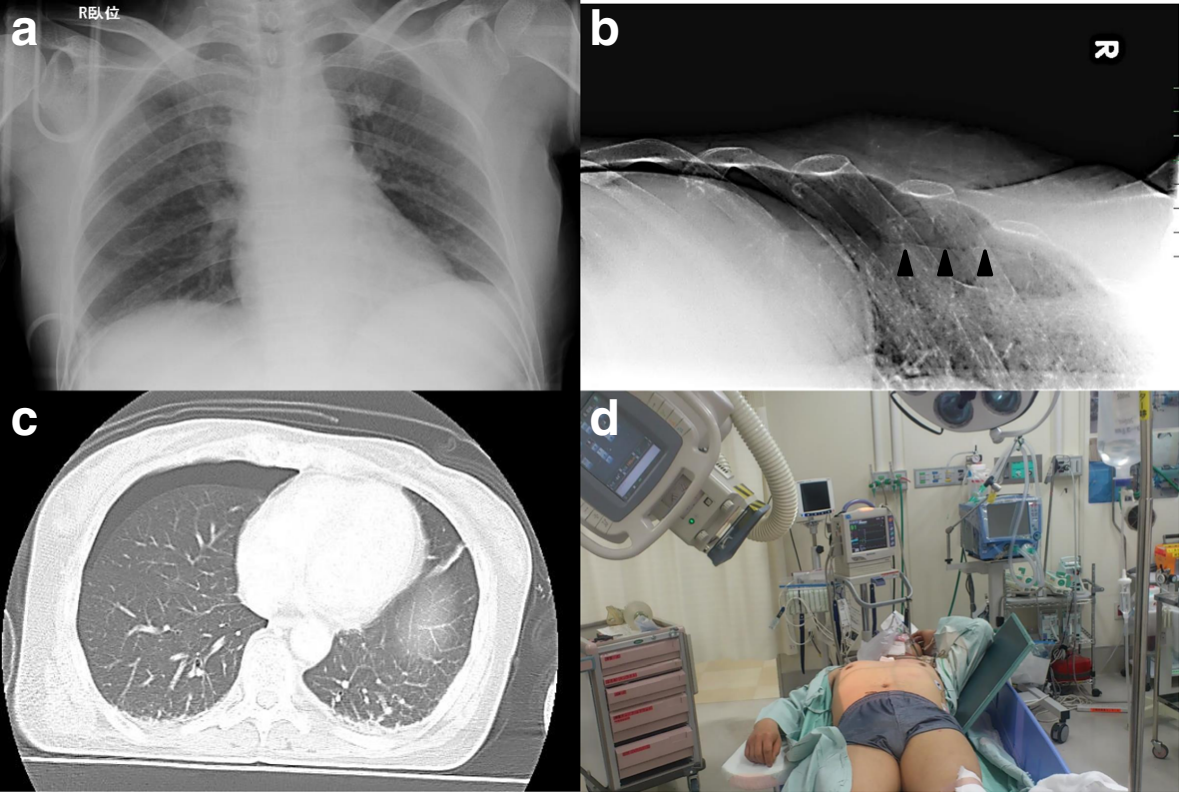
A typical case of occult pneumothorax diagnosed by OXR. This 58-year-old woman was involved in a head-on car accident and arrived with dyspnea, right chest pain. **a** Anteroposterior supine radiograph shows no abnormality. **b** OXR on the right clearly reveals a distinct visceral pleural line (arrowheads). **c** CT scan proves the existence of an occult pneumothorax on the right side. **d** Supine oblique chest radiographs could be easily performed in a trauma resuscitation area

Several studies have reported that lung ultrasonography (US) is an effective new and sensitive technique to evaluate chest trauma [3, 8–12]. However, lung US is operator dependent and operators need to learn the technique. We reported a method to detect OPX with oblique chest radiograph (OXR) without a CT scan or lung US [13] (Figs. 1 and 2). This method is simple, quick, and easily interpreted by anyone, including non-emergency, non-trauma physicians. However, the diagnostic accuracy of OXR for detecting OPX has not yet been evaluated. The aim of this study was to evaluate the usefulness of OXR in the diagnosis of OPX.

## Patients & methods

### Study design and clinical management

We conducted a prospective, noninferiority study at our emergency department in Saiseikai Yokohamashi Tobu Hospital, a tertiary-care center, from 1 January 2010 to 31 December 2014. All consecutive blunt trauma patients 18 years or older clinically suspected of OPX on arrival at the emergency department were included in this study. Clinical findings suggestive of OPX were at least one of the following conditions: (1) radiographic abnormality without overt pneumothorax finding on APXR (rib fracture, permeability decay of lung field) or (2) physical abnormalities (chest pain, bruise, subcutaneous emphysema). Exclusion criteria were overt pneumothorax, patients requiring immediate invasive interventions, transferred from another hospital, age younger than 18 years, refractory shock, cardiac arrest.

In accordance with ATLS guidelines, all patients had an examination and underwent APRX immediately upon admission. If the criteria were met, patients underwent OXR and lung US in the supine position in the emergency department, and underwent CT scans as soon as possible. OXR and lung US were performed on the bilateral-lung field as part of the routine method. In these patients, CT scans were considered the gold standard and were analyzed together with OXR and lung US. The decision to insert a chest tube for OPX was made on a case-by-case basis after a review of the images and clinical findings by attending physicians. There was no specific algorithm. This study was approved by our Institutional Scientific Board.

### Diagnosis of pneumothorax by imaging test

All OXR was performed using mobile X-ray equipment (IME-200A, Toshiba, Tokyo, Japan) before lung US and CT scan were performed. A portable film cassette was set at 45 ° against a horizontal line in suspected hemithorax. The X-ray beams were sent vertically against the cassette over the pleural interface (Fig. 1). OXR criteria for a diagnosis of pneumothorax included a distinct visceral pleural line away from the chest wall. OXR was interpreted by an emergency radiologist (F.T.) without knowledge of other information and delineated the presence of pneumothorax.

All lung US examinations were performed by attending consultant emergency physicians (M.S., T.K., T.O. and S.M.) who were board-certified in Japanese Association for Acute Medicine and trained in lung US. Theydelineated the presence of pneumothorax at the time of examination, without prior knowledge of OXR findings. A lung US imaging unit (Viamo™, Toshiba, Tokyo, Japan) with a 3.5 MHz convex probe was used. Pleural interfaces were examined at the second to fourth intercostal spaces anteriorly and the sixth to eighth spaces in the midaxillary line. Lung US diagnosis of pneumothorax was based on the previously described scanning technique (disappearance of sliding signs and loss of comet-tail artifacts at the pleural interface) [9, 12, 14], and was not assessed with Doppler function.

**Fig. 3:**
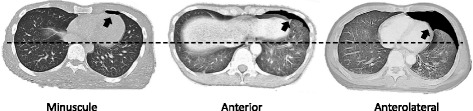
Occult pneumothorax size on CT scan classification by Wolfman et al. [15]. Minuscule pneumothorax is defined as a thin collection of air up to 1 cm thick in the greatest slice and seen on no more than 4 cm length. Anterior pneumothorax is categorized as a collection of pleural air more than 1 cm thick, located anteriorly, not extending to the mid-coronal line, and which may be seen on 4 cm or more length. Anterolateral pneumothorax is defined as pleural air extending to the mid-coronal line at least

All CT scans were performed with 64 multidetector CT scanners (Aquilion CT scanner, Toshiba, Tokyo, Japan). Immediately after emergency department resuscitation, thoracic CT scans were performed with contiguous 2 mm axial sections from the apicothorax to the symphysis pubis. The presence of pneumothorax on CT scans was judged by a supervised attending physician. Final dictated reports were reviewed by the first author (M.S.) and compared with other imaging tests. OPX size on CT was classified according to the previous report by Wolfman et al. as minuscule, anterior, or anterolateral [15] (Fig. 3).

### Statistical analyses

All statistical analyses were performed using SPSS for Windows version 15.0 (SPSS Inc., Chicago, IL, USA). Analysis was made using the chi-squared test and Fisher’s exact test as appropriate. The performance of lung US and OXR for the detection of pneumothorax was compared to CT scans as the gold standard. The diagnostic sensitivity, specificity, positive predictive value (PPV), negative predictive value (NPV), and accuracy for US and OXR were calculated using standard formulas. A value of *p* < 0.05 was considered statistically significant. The agreement between lung US and OXR was assessed using the k coefficient, which gauges whether the agreement is better than would occur by chance alone; a k value of 1 indicates perfect agreement.

## Results

### Patients suspected of occult pneumothorax

One hundred and fifty-nine patients were met the study criteria. During this study, a total of 3460 trauma patients attended the emergency department and 137 patients were diagnosed with traumatic pneumothorax. From this subset, 71 patients with traumatic pneumothorax were excluded owing to no suspicion of OPX by the attending physician, cardiopulmonary arrest or overt pneumothorax. The 159 patients underwent OXR, lung US, and CT and were included in the analysis. Of these patients, age 18 years to 99 years (median = 49.6 years), 110 (69.2 %) were men. These patients suffered from traffic accidents (71.5 %), falls (20 %), and others (8.5 %). There were 19 patients (11.9 %) who received mechanical ventilation. The average injury score was 14 ± 9.8.

### OPX diagnosed by CT gold standard and clinical management

According to the gold standard, OPX was present in 70 of these 318 thoraces (i.e., 159 patients enrolled × 2) (22.2 %). OPX involved the right side in 34 patients (52.5 %), the left side in 28 patients (42.6 %), and bilateral sides in 4 patients (4.9 %). Of the 70 OPXs, 19 were minuscule, 32 were anterior, and 19 were anterolateral. Of the 70 OPXs, 49 (70.0 %) were observed for stable cardiopulmonary condition. Sixteen OPXs underwent chest tube insertion at the time of initial care because of dyspnea, unstable condition, and disturbance of consciousness. In observed 54 OPXs, 16 (29.6 %) required a subsequent chest tube (11 of which were for progression of pneumothorax, 5 for progression of hemothorax). With a larger OPX size, the need for a chest tube increased significantly (*p* < 0.001). Viewed in terms of size (minuscule, anterior, and anterolateral) of OPX, the final chest tube insertion rate was 10.5 %, 40.6 %, and 89.5 %, respectively.

### Comparative OPX and lung US performance

**Fig. 4:**
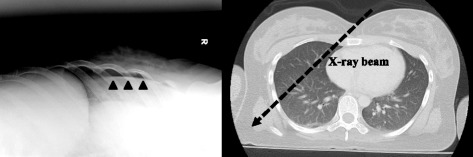
False positive case of OXR. The overlap of a large breast causes a false positive. This look like a pleural visceral line on OXR (black arrowhead)

Table 1 shows the sensitivity, specificity, PPV, NPV, and accuracy for each diagnostic method. Both methods have high specificity for OPX diagnosis but low sensitivity. There was no significant difference between the accuracies of the two methods in terms of OPX diagnosis (*p* = 0.88). The agreement between the two methods for OPX diagnosis was 93.3 % (*k* = 0.804, *p* < 0.001). Of the 318 thoraces evaluated, OXR showed a true positive result for the detection of OPXs in 43 (12.8 %), a false positive in two (0.6 %), a true negative in 246 (73.2 %), and a false negative in 27 (11.9 %). Among 43 OPXs that could be detected by OXR, 26 (61.0 %) needed tube thoracostomy. Of these 27 false negative thoraces by OXR, 16 (69.6 %) were minuscule and 21 (77.8 %) could be conservatively managed without tube thoracostomy. In the remaining six patients, conservative management failed (three for progression of hemothorax) and required a subsequent chest tube. Two false positive thorax by OXR was suspected to be due to the overlap of a large breast or skin (Fig. 4). The performance of lung US showed very similar findings to OXR. Lung US showed a true positive result for the detection of OPXs in 44 (13.8 %), a false positive in three (1 %), a true negative in 245 (77.0 %), and a false negative in 26 (8.2 %). These three false positive lung US patients had a history of tuberculous pleuritis.

**Table 1:** Performance of oblique chest radiography compared with lung ultrasonography in 158 patients with trauma

Parameter	Oblique x-ray	Lung US
Sensitivity (%)	61.4	62.9
	(0.56–0.64)	(0.57–0.66)
Specificity (%)	99.2	98.8
	(0.98–1.00)	(0.97–1.00)
Positive predictive value (%)	95.6	93.6
	(0.86–0.99)	(0.84–0.98)
Negative predictive value (%)	90.1	90.4
	(0.87–0.91)	(0.89–0.91)
Accuracy (%)	90.9	90.9
	(0.88–0.92)	(0.88–0.92)

Table 2 shows the sensitivity when viewed in terms of size of OPX for each diagnostic method. With a decrease in the size of the OPX, the sensitivity of both methods decreased significantly (*p* < 0.001). There was no significant difference in OPX size sensitivity between the two methods of OPX diagnosis.

**Table 2:** Sensitivity for detecting occult pneumothorax of oblique chest radiograph vs. lung ultrasonography viewed in terms of size of occult pneumothorax

	Occult pneumothorax size (*n* = 70)		
	Minuscule	Anterior	Anterolateral
	*n* = 19	*n* = 32	*n* = 19
Oblique X-ray sensitivity (%)	15.8	68.8	94.7
Lung US sensitivity (%)	10.5	75.0	94.7
*p*	0.63	0.59	1.0
Need for chest tube (%)	10.5	40.6	89.5

## Discussion

The currently reported incidence of OPX is between 2 and 8 % of all blunt trauma victims. The incidence of OPX among all traumatic pneumothoraces was between 52 and 72 % [3–5, 16–18]. Although pneumothorax has a high incidence in victims of major trauma, up to 76 % of all pneumothoraces may be occult to APXR interpreted by attending trauma teams in real time [19]. It was reported that most OPXs can be closely observed without a chest tube, even with positive pressure ventilation [20–22]. However, there are some OPXs that become worse as tension develops [15, 16, 23, 24]. Also, multiple traumas with high injury severity scores (ISSs) have been associated with failed observation [25, 26]. In this study, 15 of 66 patients with OPX were managed with immediate tube thoracostomy. Of the 51 patients who underwent observation, 16 (31.3 %) required subsequent chest tube insertion. Therefore, it is important that the early recognition of OPX can predict the development of tension, for which patients should be monitored and readied for chest tube insertion. CT scans detect OPX easily, but it is difficult for unstable patients to undergo a CT scan and most of these severe patients need positive pressure ventilation, which may pose a risk for progression. CT scans have the disadvantages of high cost and high doses of radiation. It is hoped that a simple method other than a CT scan or an US test for detecting OPX will attract attention.

To the best of our knowledge, this is the first study of supine OXR to detect OPX. In the primary care of trauma victims, patient movement is restricted by backboards. Upright chest radiography (CXR) is infrequently performed in the trauma bay, secondary to considerations for patient safety, especially regarding potential spine trauma and pelvic fracture. Because intrapleural air migrates into the anterior region of the pleural space (the most nondependent area) in the supine position [27], a small pneumothorax will not appear with APRX as the typical apicolateral pleural line is surrounded by intrapleural air. This means that the interface between the pneumothorax and the underlying lung is perpendicular, not parallel, to the incident x-ray beam, and cannot be easily seen. However, the x-ray beams produced by OXR could be parallel to the interface. The distribution of the intrapleural air is suitable for lung US test.

In this study, the x-ray beams of OXR were sent at 45 ° against the horizontal line. It is not certain that this angle is suitable. If the angle is close to horizontal, OXR may be able to detect smaller OPX. However, if the angle is extremely horizontal, the sternum or the other lung that overlaps an injured thorax may prevent the detection of OXR. As we considered smaller OPX to not be problematic and as the number was memorable, we defined the angle as 45 ° [13, 15].

In this study, both OXR and lung US were superior for detecting large OPXs. Other papers have reported the utility of lung US [3, 8–11]. The sensitivity in our study was lower than these previous papers. It is because there was difference in the indication criteria. Our study criteria excluded overt pneumothoraces unlike previous reports. We think that it is less important to reveal overt pneumothorax using lung US because it can be found clearly on APXR which is essential examination in primary care. Furtheremore, these previous studies were performed in far fewer centers and raised the possibility of substantial bias for individual examiners. Also, these US examiners included emergency physicians, trauma surgeons, surgical residents, and radiologist. In this study, all US examinations were performed by physicians who were trained in lung US, because not all staff were able to attain basic familiarity with lung US. Kirkpatrick and colleagues reported that US examination for detecting pneumothorax is relatively easy and the examination can be quickly learned [3]. However, there did not appear to be a learning curve over the duration of the study or differences in examiner accuracy. A good training system is essential for improving and maintaining the accuracy of US by emergency physicians and surgeons in clinical practice [28]. Although US is operator dependent, the practice and reading of OXR is simpler and easier.

In this study, the false positives of US for pneumothorax may stem from pleural adhesion with tuberculous pleuritis that were blocking lung sliding. A judgment of pneumothorax by US requires attention in Japan and developing countries where tuberculosis is more common compared to Western countries. Chest tube insertion for a patient with pleural adhesion is dangerous, especially using the Seldinger technique. Other causes of absent sliding signs include lung contusion, chronic obstructive pulmonary disease, a giant bulla, unilateral intubation, fibroid lung, and atelectasis lung [10, 14, 29, 30]. Subcutaneous emphysema is a finding suggestive of pneumothorax; however, it may produce comet-tail artifacts that have the potential of causing false negative. OXR had high specificity, however, there was two false positive case in this study. The positive finding of OXR is a collapsed lung surface (visceral pleural line) visible on X-rays. In the false positive case, a large breast was visualized by mimicking the visceral pleural line. When this false positive image of OXR was retrospectively evaluated, we could detect the presence of the lung marking distal to the false visceral pleural line. Therefore, it is important to evaluate not only the pleural visceral line but also the presence of lung marking. The majority of false negative cases of both US and OXR were minuscule pneumothorax. It is difficult to detect minuscule pneumothorax; however, this may be of low importance because most patients do not require thoracostomy.

It is important to indicate what type of patient OXR is suitable for. US is rapidly feasible following focused assessment with sonography for trauma (FAST). This is why lung US is called “Extended FAST” [3]. In this study, the majority of cases using both US and OXR could be performed within 3 min. OXR may be more laborious than US. Although OXR has a small radiological exposure compared to CT scans, patients using OXR are modestly irradiated. Therefore, it is better to limit use to patients at risk of OPX. Unfortunately, there are few useful clinical markers to predict OPX early in the resuscitation of trauma patients. Although the deep sulcus sign and the double diaphragm sign are APXR findings that suggest OPX, they lack credibility [18]. OXR may be useful to verify these subtle radiographic findings. Ball and colleagues reported that subcutaneous emphysema, pulmonary contusion, rib fractures, and female sex were independent predictors of OPX [31]. However, in a subsequent prospective study, they reported that only subcutaneous emphysema remained independently predictive of OPX [19]. There are many patients with OPX who do not have these findings. The incidence of OPX using this study’s criteria is high (33.0 %), and they may be good criteria for OXR. Unfortunately, the incidence of OPX without this study criteria is unclear. As such, further studies are needed in order to determine the indication for OXR. In reality, there may be little chance to use OXR, because the majority of trauma centers in advanced countries have US and CT. We recommend the use of OXR when CT is unusable, for example, 1) in hospitals without CT or US devices, 2) in patients in which OPX is suspected by physiological and radiological findings, 3) in emergency operation rooms and intensive care units with hemodynamically unstable patients, 4) where there is no confidence in US findings, or 5) before air transport in large fires. Our study has several limitations. First, the number of patients with OPX was small and a selection bias may have occurred because our entry criteria insufficiently covered all cases of OPX in the emergency department Second, this study did not take into account the reproducibility or inter-reader agreement of diagnostic images. It appears that inter-reader variability exists for US aside from OXR. Third, the assessment of chest tube insertion was performed by the patient’s attending physician. It is difficult to decide whether chest tube insertion is necessary or not. Additionally, attending physicians may consistently overrate the necessity of chest tube in cases of anterolateral OPX, because it is a notable finding.

## Conclusion

OXR and lung US are good tools for the diagnosis of OPX. Furthermore, the detectability of OPX by OXR may eliminate the need for an immediate tube thoracostomy. OXR is simple and effective in trauma patients who are difficult to transfer to CT scan. Although there are several methods to detect OPX, each has its distinctive characteristics, advantages, and disadvantages. The methods that best meet the needs of the situation of trauma management should be chosen.

## Abbreviations

APXR: Anteroposterior supine radiograph
CT: Computed tomography
OPX: Occult pneumothorax
US: Ultrasonography
OXR: Oblique chest radiograph
FAST: Focused assessment with sonography of trauma
NPV: Negative predictive value
PPV: Positive predictive value

## References

[1] Surgeons ACo, editor. Advanced trauma life support course for doctors. Committee on Trauma. Instructors Course Manual.

[2] Wall SD, Federle MP, Jeffrey RB, Brett CM (1983). CT diagnosis of unsuspected pneumothorax after blunt abdominal trauma. AJR Am J Roentgenol.

[3] Kirkpatrick AW, Sirois M, Laupland KB, Liu D, Rowan K, Ball CG (2004). Hand-held thoracic sonography for detecting post-traumatic pneumothoraces: the Extended Focused Assessment with Sonography for Trauma (EFAST). J Trauma.

[4] Rhea JT, Novelline RA, Lawrason J, Sacknoff R, Oser A (1989). The frequency and significance of thoracic injuries detected on abdominal CT scans of multiple trauma patients. J Trauma.

[5] Neff MA, Monk JS, Peters K, Nikhilesh A (2000). Detection of occult pneumothoraces on abdominal computed tomographic scans in trauma patients. J Trauma.

[6] Bridges KG, Welch G, Silver M, Schinco MA, Esposito B (1993). CT detection of occult pneumothorax in multiple trauma patients. J Emerg Med.

[7] In Blaosdell WF TD, editor. Trauma Management. New York: Thieme, Inc 1981

[8] Dulchavsky SA, Schwarz KL, Kirkpatrick AW, Billica RD, Williams DR, Diebel LN (2001). Prospective evaluation of thoracic ultrasound in the detection of pneumothorax. J Trauma.

[9] Kirkpatrick AW, Ng AK, Dulchavsky SA, Lyburn I, Harris A, Torregianni W (2001). Sonographic diagnosis of a pneumothorax inapparent on plain radiography: confirmation by computed tomography. J Trauma.

[10] Brook OR, Beck-Razi N, Abadi S, Filatov J, Ilivitzki A, Litmanovich D (2009). Sonographic detection of pneumothorax by radiology residents as part of extended focused assessment with sonography for trauma. J Ultrasound Med.

[11] Nagarsheth K, Kurek S (2011). Ultrasound detection of pneumothorax compared with chest X-ray and computed tomography scan. Am Surg.

[12] Soldati G, Testa A, Sher S, Pignataro G, La Sala M, Silveri NG (2008). Occult traumatic pneumothorax: diagnostic accuracy of lung ultrasonography in the emergency department. Chest.

[13] Matsumoto S, Kishikawa M, Hayakawa K, Narumi A, Matsunami K, Kitano M (2011). A method to detect occult pneumothorax with chest radiography. Ann Emerg Med.

[14] Lichtenstein D, Meziere G, Biderman P, Gepner A (1999). The comet-tail artifact: an ultrasound sign ruling out pneumothorax. Intensive Care Med.

[15] Wolfman NT, Myers WS, Glauser SJ, Meredith JW, Chen MY (1998). Validity of CT classification on management of occult pneumothorax: a prospective study. AJR Am J Roentgenol.

[16] Garramone RR, Jacobs LM, Sahdev P (1991). An objective method to measure and manage occult pneumothorax. Surg Gynecol Obstet.

[17] Tocino IM, Miller MH, Frederick PR, Bahr AL, Thomas F (1984). CT detection of occult pneumothorax in head trauma. AJR Am J Roentgenol.

[18] Ball CG, Kirkpatrick AW, Fox DL, Laupland KB, Louis LJ, Andrews GD (2006). Are occult pneumothoraces truly occult or simply missed?. J Trauma.

[19] Ball CG, Ranson K, Dente CJ, Feliciano DV, Laupland KB, Dyer D (2009). Clinical predictors of occult pneumothoraces in severely injured blunt polytrauma patients: A prospective observational study. Injury.

[20] Brasel KJ, Stafford RE, Weigelt JA, Tenquist JE, Borgstrom DC (1999). Treatment of occult pneumothoraces from blunt trauma. J Trauma.

[21] Moore FO, Goslar PW, Coimbra R, Velmahos G, Brown CV, Coopwood TB (2011). Blunt traumatic occult pneumothorax: is observation safe?--results of a prospective, AAST multicenter study. J Trauma.

[22] Mowery NT, Gunter OL, Collier BR, Diaz JJ, Haut E, Hildreth A (2011). Practice management guidelines for management of hemothorax and occult pneumothorax. J Trauma.

[23] Enderson BL, Abdalla R, Frame SB, Casey MT, Gould H, Maull KI (1993). Tube thoracostomy for occult pneumothorax: a prospective randomized study of its use. J Trauma.

[24] Collins JC, Levine G, Waxman K (1992). Occult traumatic pneumothorax: immediate tube thoracostomy versus expectant management. Am Surg.

[25] Hill SL, Edmisten T, Holtzman G, Wright A (1999). The occult pneumothorax: an increasing diagnostic entity in trauma. Am Surg.

[26] Barrios C, Tran T, Malinoski D, Lekawa M, Dolich M, Lush S (2008). Successful management of occult pneumothorax without tube thoracostomy despite positive pressure ventilation. Am Surg.

[27] Ball CG, Kirkpatrick AW, Laupland KB, Fox DL, Litvinchuk S, Dyer DM (2005). Factors related to the failure of radiographic recognition of occult posttraumatic pneumothoraces. Am J Surg.

[28] Emergency ultrasound guidelines. Ann Emerg Med. 2009 Apr;53(4):550–70. 10.1016/j.annemergmed.2008.12.013 19303521

[29] Rowan KR, Kirkpatrick AW, Liu D, Forkheim KE, Mayo JR, Nicolaou S (2002). Traumatic pneumothorax detection with thoracic US: correlation with chest radiography and CT--initial experience. Radiology.

[30] Lichtenstein DA, Lascols N, Prin S, Meziere G (2003). The “lung pulse”: an early ultrasound sign of complete atelectasis. Intensive Care Med.

[31] Ball CG, Kirkpatrick AW, Laupland KB, Fox DI, Nicolaou S, Anderson IB (2005). Incidence, risk factors, and outcomes for occult pneumothoraces in victims of major trauma. J Trauma.

